# Supplementary material for: Evaluation of pharmaceutically compounded oral caffeine on the impact of medication adherence and risk of readmission among preterm neonates: A single-center quasi-experimental study
Source: PLoS One. 2022 Nov 9;17(11):e0275655. doi: 10.1371/journal.pone.0275655 (PMC9645656; doi:10.1371/journal.pone.0275655)
Supplement: Attachment — Submitted filename: Response to Reviewers.docx [file pone.0275655.s002.docx]

Dear Editor and reviewers

Thank you for your appreciation, comments, and guidance that really helped us to enhance our manuscript. We tried our best to work on it and address each comment. Looking for your kind response.

Regards

Dr. Gul Ambreen

| Editor and Reviewer Comments: | Response |
| --- | --- |
| Journal Requirements: |  |
| Please ensure that your manuscript meets PLOS ONE's style requirements, including those for file naming. The PLOS ONE style templates can be found at  [https://journals.plos.org/plosone/s/file?id=wjVg/PLOSOne_formatting_sample_main_body.pdf](https://eur03.safelinks.protection.outlook.com/?url=https%3A%2F%2Fjournals.plos.org%2Fplosone%2Fs%2Ffile%3Fid%3DwjVg%2FPLOSOne_formatting_sample_main_body.pdf&data=05%7C01%7Cgul.ambreen%40aku.edu%7C443fa18f02b24f0a552308da547e2cac%7Ca5d4252a02f94e6096f09733baae4919%7C0%7C0%7C637915198294633950%7CUnknown%7CTWFpbGZsb3d8eyJWIjoiMC4wLjAwMDAiLCJQIjoiV2luMzIiLCJBTiI6Ik1haWwiLCJXVCI6Mn0%3D%7C3000%7C%7C%7C&sdata=B7E7SrSl7x8bSHtf%2F7Y7BtGTY6HR7JL5s6CbU%2BxTbeI%3D&reserved=0)  and  [https://journals.plos.org/plosone/s/file?id=ba62/PLOSOne_formatting_sample_title_authors_affiliations.pdf](https://eur03.safelinks.protection.outlook.com/?url=https%3A%2F%2Fjournals.plos.org%2Fplosone%2Fs%2Ffile%3Fid%3Dba62%2FPLOSOne_formatting_sample_title_authors_affiliations.pdf&data=05%7C01%7Cgul.ambreen%40aku.edu%7C443fa18f02b24f0a552308da547e2cac%7Ca5d4252a02f94e6096f09733baae4919%7C0%7C0%7C637915198294790201%7CUnknown%7CTWFpbGZsb3d8eyJWIjoiMC4wLjAwMDAiLCJQIjoiV2luMzIiLCJBTiI6Ik1haWwiLCJXVCI6Mn0%3D%7C3000%7C%7C%7C&sdata=cggpt83%2BaO7XeUWLsSaCt1lWspy0YwDQ2WzC7Kk3PnU%3D&reserved=0) | Manuscript is checked to ensure manuscript meets PLOS ONE's style requirements, including those for file naming. Also checked The PLOS ONE style templates and made the changes as required  And uploaded the figures in required format |
| Please ensure that you include a title page within your main document. We do appreciate that you have a title page document uploaded as a separate file, however, as per our author guidelines (<http://journals.plos.org/plosone/s/submission-guidelines#loc-title-page>) we do require this to be part of the manuscript file itself and not uploaded separately.  Could you therefore please include the title page into the beginning of your manuscript file itself, listing all authors and affiliations. | We included the title page into the beginning of our manuscript file listing all authors and affiliations. |
| We note that you have indicated that data from this study are available upon request. PLOS only allows data to be available upon request if there are legal or ethical restrictions on sharing data publicly. For more information on unacceptable data access restrictions, please see [http://journals.plos.org/plosone/s/data-availability#loc-unacceptable-data-access-restrictions](https://eur03.safelinks.protection.outlook.com/?url=http%3A%2F%2Fjournals.plos.org%2Fplosone%2Fs%2Fdata-availability%23loc-unacceptable-data-access-restrictions&data=05%7C01%7Cgul.ambreen%40aku.edu%7C443fa18f02b24f0a552308da547e2cac%7Ca5d4252a02f94e6096f09733baae4919%7C0%7C0%7C637915198294790201%7CUnknown%7CTWFpbGZsb3d8eyJWIjoiMC4wLjAwMDAiLCJQIjoiV2luMzIiLCJBTiI6Ik1haWwiLCJXVCI6Mn0%3D%7C3000%7C%7C%7C&sdata=rTNgiXsWF4LU0suduplExGbYoKTqVc32bqLlkHzP%2Brc%3D&reserved=0).  In your revised cover letter, please address the following prompts:  a) If there are ethical or legal restrictions on sharing a de-identified data set, please explain them in detail (e.g., data contain potentially sensitive information, data are owned by a third-party organization, etc.) and who has imposed them (e.g., an ethics committee). Please also provide contact information for a data access committee, ethics committee, or other institutional body to which data requests may be sent.  b) If there are no restrictions, please upload the minimal anonymized data set necessary to replicate your study findings as either Supporting Information files or to a stable, public repository and provide us with the relevant URLs, DOIs, or accession numbers. For a list of acceptable repositories, please see <http://journals.plos.org/plosone/s/data-availability#loc-recommended-repositories>.  We will update your Data Availability statement on your behalf to reflect the information you provide. | We have revised and uploaded the cover letter and mentioned the reason for data sharing statement. |
| Thank you for stating the following financial disclosure:  “Muhammad Shahzad Aslam received this award. This work was supported by the Research Management Center, Xiamen University Malaysia, grant number XMUMRF/2020-C6/ITCM/0005.”  Please state what role the funders took in the study.  If the funders had no role, please state: "The funders had no role in study design, data collection and analysis, decision to publish, or preparation of the manuscript."  If this statement is not correct you must amend it as needed.  Please include this amended Role of Funder statement in your cover letter; we will change the online submission form on your behalf. | We included the amended Role of Funder statement in our cover letter; please change the online submission form on our behalf. |
| Thank you for stating the following in the Competing Interests section:  “Muhammad Shahzad Aslam is the academic editor in PLOS ONE .  All other authors declare that they have no conflict of interest.”  Please confirm that this does not alter your adherence to all PLOS ONE policies on sharing data and materials, by including the following statement: "This does not alter our adherence to  PLOS ONE policies on sharing data and materials.” (as detailed online in our guide for authors <http://journals.plos.org/plosone/s/competing-interests>).  If there are restrictions on sharing of data and/or materials, please state these. Please note that we cannot proceed with consideration of your article until this information has been declared.  Please include your updated Competing Interests statement in your cover letter; we will change the online submission form on your behalf. | We have included our updated Competing Interests statement in revised cover letter;  Please change the online submission form on our behalf. |
| Please review your reference list to ensure that it is complete and correct. If you have cited papers that have been retracted, please include the rationale for doing so in the manuscript text, or remove these references and replace them with relevant current references. Any changes to the reference list should be mentioned in the rebuttal letter that accompanies your revised manuscript. If you need to cite a retracted article, indicate the article’s retracted status in the References list and also include a citation and full reference for the retraction notice. | Checked for this matter |
| **Reviewer #1: GENERAL COMMENTS** The findings of the research are clearly presented and add to the body of knowledge. The submission should be accepted after minor corrections. ABSTRACT Well-written. Preferably, arrange the keywords in alphabetical order.  BACKGROUND Page 3 (line 24) and Page 4 (Line 1): The last sentence lacks fluidity.  METHODS Page 7, Line 6: Data collection and … Page 8, Line 10 – 12: Sentence is ambiguous. Rephrase.  RESULTS Well-written.  DISCUSSION Well-written.  CONCLUSION Apt.  REFERENCES The authors need to re-visit the References and ensure there is consistency with the Journal’s guidelines.  TABLES AND FIGURES Tables that have p-values should include the level of significance as a footnote. | Thank you for the comments  Note: pg and line numbers are changed as made few changes in manuscript- but highlighted red in the file named 'Revised Manuscript with Track Changes'  ABSTRACT keywords are rearranged in alphabetical order.  BACKGROUND Page 3 (line 24) and Page 4 (Line 1) are rephrased  METHODS  Page 7, Line 6: …. Changed to outcome measures and data collection  Page 8, Line 10 – 12: Sentence is Rephrased.  RESULTS, DISCUSSION and CONCLUSION thank you  REFERENCES References are checked to ensure the consistency with the Journal’s guidelines.  TABLES AND FIGURES statement for the p-values is mentioned as a footnote for tables where needed. |
| Reviewer #2:  Overall well written report. Kudos to authors for a good job done. Minor changes suggested |  |
| Page 1 Line 6: suggest replace "until corrected" with "in neonates of" Line 8-9: Suggest replace sentence with "Pharmaceutically compounded oral caffeine (PCC) could be an appropriate alternate dosage form" Line 20: replace "added" with "included" Line 24: pre-phase refills are higher than post-phase refill?  Page 2 Line 10: replace with "in other Resource-limited settings"  Page 7 Line 2: replace "was" with "were" Line 6.: Replace Date with Data  Results Give subheadings to separate the various sections for better clarity.  Page 10 line 18 Caffeine refills pre-PCC has been swapped with Post PCC making it seem refills for caffeine were higher pre-phase than post-phase which is not the case in table 2. Kindly reconcile.  Discussion Page 11 Line 24: replace "results in" with " , resulting in"  page 12 Line 2: Kindly avoid making statements along the lines of being the first which may be refuted in future. May replace with "To the best of our knowledge, this is first study addressing........"  Line 19, "needed required" replace with either one of them place comma (,) between MV and PCC Suggest sentence be split into two or more to improve clarity. from Line 18 to 22  Page 13 line 5-6 Move sentence to limitations section as strengths and limitations of study | Page 1 Line 6: replaced "until corrected" with "in neonates of" Line 8-9: replaced sentence with "Pharmaceutically compounded oral caffeine (PCC) could be an appropriate alternate dosage form" Line 20: replaced "added" with "included" Line 24: corrected “77.6% pre-phase vs 97.5% post-phase”  Page 2 Line 10: replaced with "in other Resource-limited settings"  Page 7 Line 2: replaced "was" with "were" Line 6.: Replaced Date with Data  Results Given subheadings and separated the various sections for better clarity.  Page 10 line 18 Corrected to 77.6% pre-phase vs 97.5% post-phase  Discussion Page 11 Line 24: replaced "results in" with " , resulting in"  page 12 Line 2: made the changes as guided and rephrased as "To the best of our knowledge, this is first study addressing........"  Line 19, "needed required" replaced with needed  sentence split into two to improve clarity. from Line 18 to 22  Page 13 line 5-6 Moved sentence to limitations section as strengths and limitations of study  Line 16: checked section discussion for this matter and rephrased where needed. |
